# Supplementary material for: Contribution of the Type III Secretion System (T3SS2) of Vibrio parahaemolyticus in Mitochondrial Stress in Human Intestinal Cells
Source: Microorganisms. 2024 Apr 17;12(4):813. doi: 10.3390/microorganisms12040813 (PMC11051933; doi:10.3390/microorganisms12040813)
Supplement: Supplementary file 1 [file microorganisms-12-00813-s001.zip › microorganisms-2940770-supplementary.pdf]

Supplementary Figure S1.

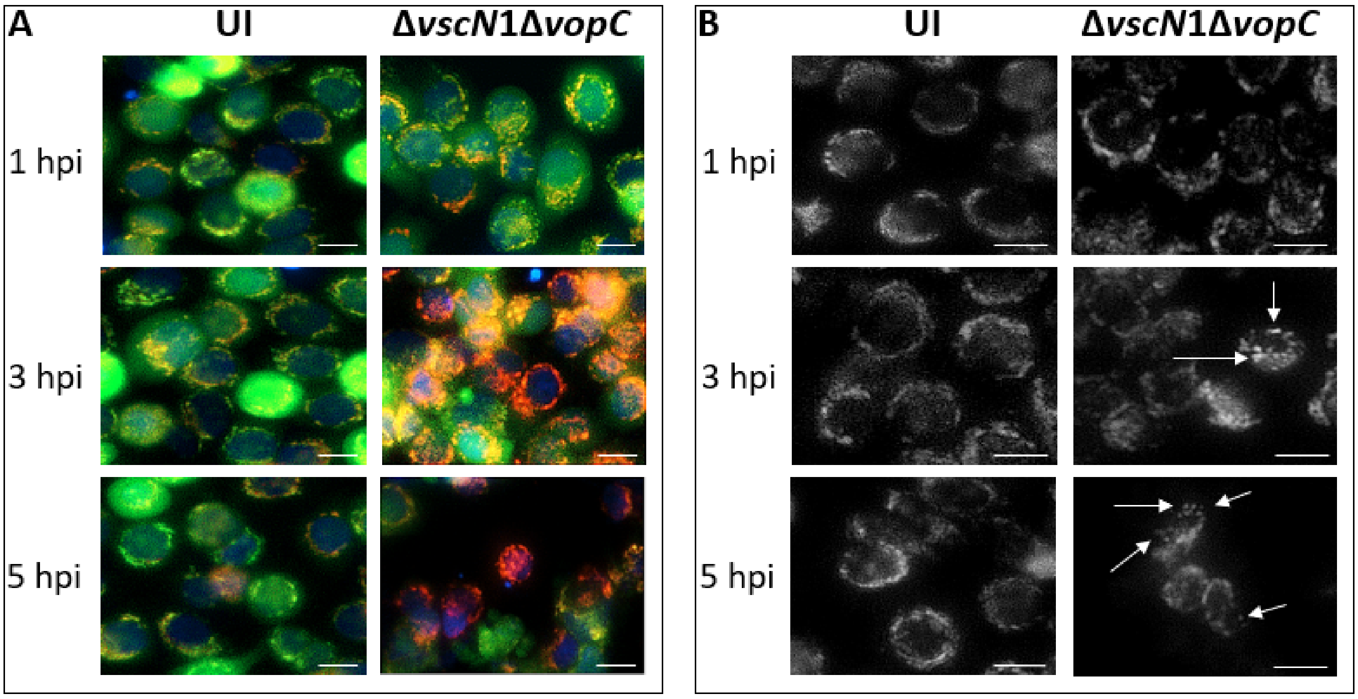

Supplementary Figure S1. *V. parahaemolyticus vopC* mutant strain induce mPTP opening and mitochondrial fragmentation in intestinal cells T3SS2-dependent. (**A**) Uninfected HT-29 cells (UI) and infected with *V. parahaemolyticus* Δ*vscn*1 Δ*vopC* strain (Δ*vopC*, T3SS2+) at 1, 3 and 5 hpi. In the figures, green color indicates the mPTP are closed (healthy mitochondria), red color shows the mitochondrial mass (stress condition), and blue color represents the Hoechst-stained nuclei. (**B**) Mitochondria network morphology at 1, 3 and 5 hpi was analyzed by microscopy. White arrows show cells with mitochondrial fragmentation. Representative images from 3 independent trials are shown. Scale bar: 10μm.
